# Effects of Elevated Carbon Dioxide, Elevated Temperature, and Rice Growth Stage on the Community Structure of Rice Root–Associated Bacteria

**DOI:** 10.1264/jsme2.ME14011

**Published:** 2014-05-31

**Authors:** Takashi Okubo, Takeshi Tokida, Seishi Ikeda, Zhihua Bao, Kanako Tago, Masahito Hayatsu, Hirofumi Nakamura, Hidemitsu Sakai, Yasuhiro Usui, Kentaro Hayashi, Toshihiro Hasegawa, Kiwamu Minamisawa

**Affiliations:** 1 Graduate School of Life Sciences, Tohoku University, Katahira, Aoba-ku, Sendai, Miyagi 980–8577, Japan; 2 National Institute for Agro-Environmental Sciences, 3–1–3 Kannondai, Tsukuba, Ibaraki 305–8604, Japan; 3 Memuro Research Station, National Agricultural Research Center for Hokkaido Region, Shinsei, Memuro-cho, Kasaigun, Hokkaido 082–0081, Japan; 4 Taiyo-Keiki Co., Ltd., 1–12–3 Nakajujo, Kita-ku, Tokyo, 114–0032, Japan

**Keywords:** carbon dioxide, methane, plant-associated bacteria, rice, warming

## Abstract

The effects of free-air carbon dioxide enrichment (FACE) and elevated soil and water temperature (warming) on the rice root–associated bacterial community were evaluated by clone library analysis of the 16S ribosomal RNA gene. Roots were sampled at the panicle initiation and ripening stages 41 and 92 days after transplanting (DAT), respectively. The relative abundances of the methanotrophs *Methylosinus* and *Methylocystis* were increased by warming and decreased by FACE at 92 DAT, which indicated that microbial methane (CH_4_) oxidation in rice roots may have been influenced by global warming. The relative abundance of *Burkholderia kururiensis* was increased by warming at 41 DAT and by FACE or warming at 92 DAT. The abundances of methanotrophs increased during rice growth, which was likely induced by an enhancement in the emission of CH_4_ from the paddy fields, suggesting that CH_4_ is one of the predominant factors affecting the structure of the microbial community in rice roots. Marked variations in the community structure were also observed during rice growth in other genera: *Bradyrhizobium*, *Clostridium*, and an unknown genus close to *Epsilonproteobacteria* were abundant at 92 DAT, whereas *Achromobacter* was abundant at 41 DAT. These results demonstrated that the community structures of rice root-associated bacteria were markedly affected by FACE, temperature, and the rice growth stage.

The atmospheric carbon dioxide concentration ([CO_2_]) was stable at 270 ppm for at least 1,000 years prior to the start of the industrial revolution. Since that time, [CO_2_] has been rising and has reached nearly 400 ppm (7). The increase in [CO_2_] is expected to enhance the growth and yield of C3 crops, including rice (29). Previous studies reported that increases in [CO_2_] quantitatively and qualitatively altered the release of labile sugars, organic acids, and amino acids from plant roots (2, 6), which may influence the activity of rhizospheric and root–associated microbes, including methanogenesis (18). Tokida *et al.* (32, 33) reported that the emission of CH_4_ from paddy fields was significantly increased by [CO_2_] and/or temperature elevations. CH_4_ generated in soil is diffused into rice roots, transported to the shoot via aerenchyma, and finally released from micropores in the leaf sheaths (25). The rhizosphere and rice roots were previously shown to be the main areas CH_4_ oxidation in rice paddies (4) because the oxidation of CH_4_ was inactive in flooded soils without oxygen derived from rice roots.

Although the responses of rice plants to elevated atmospheric [CO_2_] and/or temperature have been studied in detail, those of plant-associated microbes remain unknown (29, 33). The responses of plant-associated microbes to global climate changes may potentially be important because they play major roles in the flow of carbon and nitrogen as the primary utilizers of plant-derived compounds in the rhizosphere.

A technique to enrich bacterial cells obtained from plant tissues has provided a gateway to access plant-associated bacterial communities (14), and this has facilitated a deeper understanding of microbial community shifts caused by environmental factors (15, 27). In the present study, we investigated the community structure of rice root–associated bacteria in environments with elevated temperature (ET) and/or [CO_2_] using the bacterial cell enrichment method (14). Our results have provided an insight into carbon and nitrogen cycles in rice paddies under a changing climate.

## Materials and Methods

### Study site, [CO_2_] enrichment, and soil and water warming

The free-air carbon dioxide enrichment (FACE) and soil warming experiments were conducted in a rice paddy field at Tsukubamirai, Ibaraki, Japan (35°58′27″N, 139°59′32″E, 10 m above sea level), during the 2011 growing season. The soil there is a Fluvisol, which is typical of alluvial areas. The bulk density was 0.87 Mg m^−3^, and total C and N were 21.4 mg g^−1^ and 1.97 mg g^−1^, respectively. The cation exchange capacity was 202 μmolc g^−1^ (12). The experimental site was established in 2010, and control protocols for the FACE and warming treatments were described previously (23, 32). Briefly, four rice paddy fields were used as replicates, each with areas at ambient [CO_2_] (AMBI) and also at enriched [CO_2_] (FACE) with a target concentration of 200 μmol mol^−1^ above AMBI. Each treatment area was a 240-m^2^ octagon (“ring,” hereafter). The FACE rings used emission tubes on all eight sides at a height of approximately 30 cm above the canopy, and these released pure CO_2_ from wind-ward sides to maintain a stable concentration at the center of the rings (ambient + 200 μmol mol^−1^). The AMBI and FACE rings were separated by at least 70 m (center to center), which was previously shown to be sufficient to prevent cross-contamination by CO_2_ from a FACE ring (13).

Warming treatments were also conducted by a split-plot design in each ring with two levels of soil and water temperatures: normal temperature (NT) and ET with the target of 2°C above NT. Warming was achieved using heating wires placed on the soil surface between the rows, with the water temperature continuously measured by a Pt100 thermometer (Chino Co. Ltd., Tokyo, Japan). The water and plow layer (at a depth of 10 cm) temperatures were almost uniformly elevated. The ET plot was enclosed using corrugated PVC panels to prevent an exchange of the paddy water with the surrounding area.

### Rice cultivation and fertilization

Rice (*Oryza sativa* L. cv. Koshihikari) was sown on 25 April 2011 in seedling trays with 448 cells (Minoru Pot 448, Minoru Industrial Co., Ltd., Okayama, Japan). Three pre-germinated seeds were planted in each cell of the tray. After emergence, we raised seedlings in a puddled open field with a tunnel cloche or floating mulch for the first 2 weeks. On 25 and 26 May, seedlings at the five-leaf stage were transplanted into the rings by hand, with three seedlings per hill. Hills and rows were 15 and 30 cm apart, respectively, with a resultant density of 22.2 hills m^−2^. Fertilizers were applied as a basal dressing. Nitrogen was supplied at 8 g N m^−2^ (2 and 6 g N m^−2^ as urea and coated urea, respectively; 4 g of LP-100 and 2 g of LP-140; JCAM-Agri Co., Ltd., Tokyo, Japan). Phosphate and potassium were applied as a compound fertilizer (Sumitomo Chemical Co., Ltd., Tokyo, Japan) containing 4.4 g P m^−2^ and 8.3 g K m^−2^, respectively. The method for rice cultivation and fertilization was described previously (12).

### CH_4_ emission measurements

The emission of CH_4_ was measured weekly or biweekly between 7 June and 23 August using a closed chamber method, as described previously (16). Each chamber consisting of lower (60 cm H) and upper (60 cm H) sections was placed over 4 hills of rice plants with a basal area of 30 × 60 cm. The upper section of the chamber fit over the lower one and was supported by a water-filled groove surrounding the outer top lip of the lower section, thereby providing an airtight seal between the two sections and surrounding atmosphere. Gas samples were collected from the chamber 0, 10, and 20 min after placement of the chamber. The samples were injected into pre-evacuated 19-mL glass vials and brought back to the laboratory for analysis. The mixing ratio of CH_4_ was determined by gas chromatography equipped with a flame ionization detector (GC-14B; Shimazu, Kyoto, Japan). The emission of CH_4_ was calculated based on an increase in the mixing ratio of the basal area of the chamber, chamber volume, and temperature inside the chamber. An analysis of variance (ANOVA) was conducted on the cumulative amount of CH_4_ that was emitted during 41–90 days after transplanting (DAT) using a general linear procedure. [CO_2_], temperature, and [CO_2_]×temperature were treated as fixed effects, while ring and ring×[CO_2_] were treated as random effects.

### Rice sampling

Three whole rice plants were carefully dug out by hand from each treatment plot on 5 July 2011 (41 d after transplanting [DAT]), which corresponded to the panicle initiation stage, and 25 August (92 DAT), which corresponded to the ripening stage (2 CO_2_ treatments [AMBI or FACE] × 2 temperature treatments [ET or NT] × 4 rings × 2 growth stages). A block of plow-layer soil, 30 × 15 × 30 cm (length × width × depth), was also taken with the plants. Whole rice plants were then immediately transported to the laboratory and washed with tap water to remove adhering soil particles. The roots were separated from the shoot and stored at −80°C until they were used for analysis.

### DNA preparation and clone library construction

The root samples were manually ground into a fine powder in liquid nitrogen using a mortar and pestle. Three ground root samples, collected from the same ring of the same treatment, were composited and then homogenized again in a blender. The composited samples were subjected to DNA extraction by the bacterial cell enrichment method (14), PCR amplification, and clone library analysis targeting the 16S rRNA gene (14). Briefly, bacterial DNA was extracted from the composited roots using the bacterial cell enrichment method (14). The PCR clone libraries for 16S rRNA genes were constructed as follows: 10 ng total bacterial DNA was used as a template in a final reaction volume of 50 μL, including 0.1 μM of each primer and 2 U of Ex Taq DNA polymerase (Takara Bio, Otsu, Japan) with the universal primers 27F (5′-AGAGTTTGAT CMTGGCTCAG-3′) and 1525R (5′-AAGGAGGTGWTCCARCC-3′) (17). The cycling conditions were an initial denaturation step of 2 min at 94°C; 25 cycles of 30 s at 94°C, 30 s at 55°C, and 1.5 min at 72°C; and a final extension step of 8 min at 72°C. PCR products of the predicted size (1500 bp) were purified using a Wizard SV Gel and PCR Clean-Up System (Promega Japan, Tokyo, Japan). Four composited samples, collected from four rings within the same treatment, were combined, then ligated into the pGEM-T Easy plasmid vector (Promega Japan) at 25°C for 1 h. A partial sequence of the 16S rRNA gene was determined by Takara Bio Inc. (Otsu, Japan) using the 27F forward primer as a sequencing primer.

### Bioinformatics analysis

Low-quality and contaminated reads were removed using OrientationChecker (1). A partial 16S rRNA gene sequence (corresponding to bases 109 to 684 of the *Escherichia coli* 16S rRNA gene) was used for sequence analyses. Chimeric sequences were detected using MALLARD (1). The remaining sequences were aligned using CLUSTAL W (31). Based on the alignment, a distance matrix was constructed using the DNADIST program from PHYLIP ver. 3.66 (http://evolution.genetics.washington.edu/phylip.html) with the default parameters. The resulting matrix was clustered using Mothur (28) to generate diversity indexes with a threshold of a 97% sequence identity. Library coverage was calculated with the nonparametric estimator *C* (9). UniFrac (19) was applied with the abundance-weighted option to examine similarities among clone libraries.

The phylogenetic composition of the library sequences was evaluated using the RDPmultiple classifier (34), with confidence levels of 80%. Sequences assigned to *Burkholderia* and *Bradyrhizobium* were extracted separately and aligned with reference sequences using CLUSTAL W (31). Neighbor-joining trees were constructed using MEGA version 5.1 (30), and 1,000 bootstrap replicates were used to generate a consensus tree.

### Nucleotide sequence accession numbers

The nucleotide sequences of 16S rRNA genes in the clone libraries have been deposited in DDBJ under the accession numbers shown in Table 1.

## Results and Discussion

### Overview of bacterial community structures

The statistics of the clone libraries are summarized in Table 2. In all treatments, the number of operational taxonomic units (OTUs) and the Chao1 and Shannon diversity indexes were greater at 92 DAT than at 41 DAT. These indexes were decreased in the samples at 92 DAT due to the elevation in [CO_2_] or soil and water temperature. No clear trend was observed in the samples at 41 DAT.

**Fig. 1:**
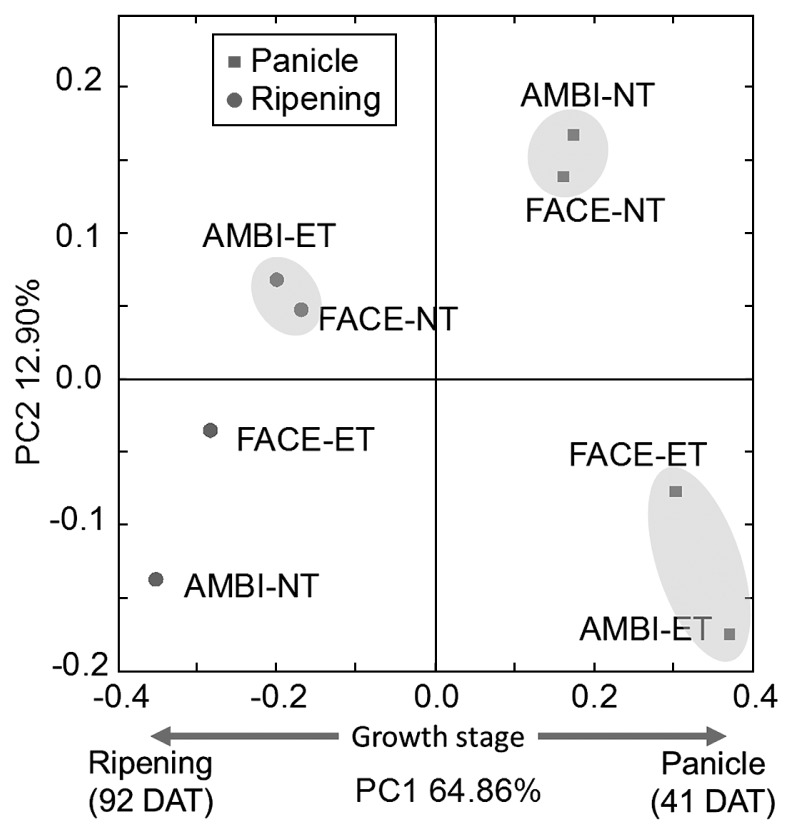
Principal coordinate analysis of the 16S rRNA gene libraries of bacterial communities in rice roots under normal and elevated [CO_2_] and temperature conditions. The ordination was constructed using UniFrac distances weighted by the relative abundances. Principal component 1 (PC1) and principal component 2 (PC2) are plotted on the *x*- and *y*-axes, respectively. The percentage of variation explained by the plotted principal coordinates is indicated on the axes. Samples were collected at the panicle initiation stage (●, 41 DAT) and ripening stage (■, 92 DAT).

Principal coordinate analysis was performed using all sequence data (Fig. 1) in order to obtain an overview of bacterial community shifts caused by the rice growth stage and elevation in [CO_2_] and temperature. Samples were clearly separated along the first principal component (PC1) axis (64.86%) according to the rice growth stage, which indicated that bacterial community structures markedly changed as the host plant grew. Two tight clusters were formed according to the temperature condition in samples at 41 DAT, suggesting that community structures were more sensitive to the temperature change than to that of [CO_2_] at 41 DAT. Community shifts in samples at 92 DAT were more complicated. The degree of the community shift from the control (AMBI-NT) was smaller in the simultaneous treatment (FACE-ET) than in the other treatments (FACE-NT and AMBI-ET). Furthermore, FACE-NT and AMBI-ET were clustered close to each other at 92 DAT.

### Phylogenetic composition

In all treatments, the abundances of *Methylosinus* and *Methylocystis* were markedly higher at 92 DAT (5.0–15.3%) than at 41 DAT (0.0–1.1%) (Table 3). At the beginning of the rice growing period, the amount of CH_4_ emitted was very low (Table 4). As the season progressed, it steadily increased to approximately 14–18 mg C-CH_4_ m^−2^ h^−1^ at 48 DAT (July 12), and a high emission level of CH_4_ was maintained until 83 DAT (August 16). These results suggest that rice roots were exposed to a large amount of CH_4_ between the first (41 DAT) and second sampling (92 DAT), and this may have caused the increase observed in the relative abundances of *Methylosinus* and *Methylocystis* in the rice roots at 92 DAT. The cumulative emission of CH_4_ during 41–90 DAT was the highest in FACE-ET (Table 4). However, the relative abundances of *Methylosinus* and *Methylocystis* in FACE-ET were intermediate among the four treatments (Table 3), which suggested that factors other than CH_4_ also affected the relative abundances of methanotrophs. At 92 DAT, the relative abundances of *Methylosinus* and *Methylocystis* were increased by the elevation in temperature in both AMBI and FACE plots, but were decreased by that in [CO_2_]. A previous FACE experiment conducted in Japan showed that nitrogen concentrations in rice plants were decreased by elevations in [CO_2_] (29), which was at least partially attributed to a dilution effect due to the greater production of dry matter. Many studies have suggested the stimulatory effect of nitrogen on CH_4_ oxidation in rice paddies (3). Such a change in the nitrogen condition may affect the activities of rice root–associated methanotrophs, leading to decreases in the relative abundance by elevations in [CO_2_]. Tokida *et al.* (32) previously reported that the emission of CH_4_ from paddy fields was significantly increased by elevations in [CO_2_], and this effect was considered to be mainly derived from an increase in rhizodeposition. However, our results suggest that one reason for the increase in CH_4_ emission with elevations in [CO_2_] may have been a decline in the abundance of methanotrophs associated with rice roots. An enhancement in CH_4_ oxidation activity in rice roots is vital for breaking the positive feedback loop of CH_4_ emission that will occur with increases in atmospheric [CO_2_] in the future. However, we did not observe a clear increase in the emission of CH_4_ by elevations in [CO_2_] under the NT condition (Table 4). Therefore, the effects of [CO_2_] and temperature elevation on the oxidation activity of CH_4_ in paddy fields need to be studied in more detail.

**Fig. 2:**
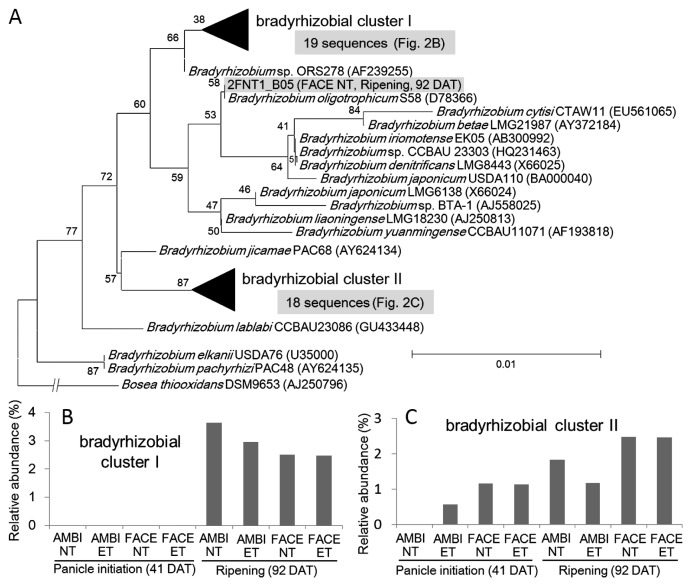
Phylogenetic position and distribution of rice root–associated *Bradyrhizobium*. (A) Phylogenetic tree of rice root–associated bradyrhizobia extracted from the clone libraries. (B, C) Distributions of reads belonging to the two large groups in panel (A). Accession numbers are shown following the strain names.

Members of the genus *Bradyrhizobium* are important nitrogen-fixing bacteria in rice roots (5). In all treatments, the relative abundance of *Bradyrhizobium* was greater at 92 DAT (4.1–5.6%) than at 41 DAT (0.0–1.2%) (Table 3). However, no apparent effects of the elevation in [CO_2_] and temperature were observed at 41 or 92 DAT. To perform a detailed phylogenetic analysis of rice root–associated *Bradyrhizobium*, 16S rRNA reads assigned to *Bradyrhizobium* were extracted from clone libraries and a phylogenetic tree was constructed with the other members of bradirhizobia (Fig. 2A). Rice root–associated bradyrhizobia were clustered into two groups that were phylogenetically close to *Bradyrhizobium* sp. ORS278 (bradyrhizobial cluster I) (8, 26) and *Bradyrhizobium jicamae* (bradyrhizobial cluster II). Bradyrhizobial cluster I was only observed at 92 DAT (Fig. 2B), whereas bradyrhizobial cluster II was observed at both 41 and 92 DAT (Fig. 2C). In a previous study, *Bradyrhizobium* sp. ORS278 was reported to colonize the surface and intercellular space of rice roots and also fix nitrogen (5). Our clone library analysis suggested that members of bradyrhizobial cluster I may be representative nitrogen-fixing bacteria in the rice root at the ripening stage. The relative abundance of bradyrhizobial cluster I (Fig. 2B) was strongly correlated with those of *Methylosinus* and *Methylocystis* (Pearson’s correlation coefficient, *r*=0.83, *P*=0.011; *r*=0.92, *P*=0.001, respectively). One explanation for this correlation was the possible metabolic coupling by which one-carbon compounds oxidized by methanotrophs such as methanol may be partially consumed by these *Bradyrhizobium* species. *Bradyrhizobium* sp. ORS278 (bradyrhizobial cluster I) has a gene for methanol oxidation (BRADO5483–BRADO5487) on the genome, and was able to oxidize methanol (Seki *et al.* unpublished result).

**Fig. 3:**
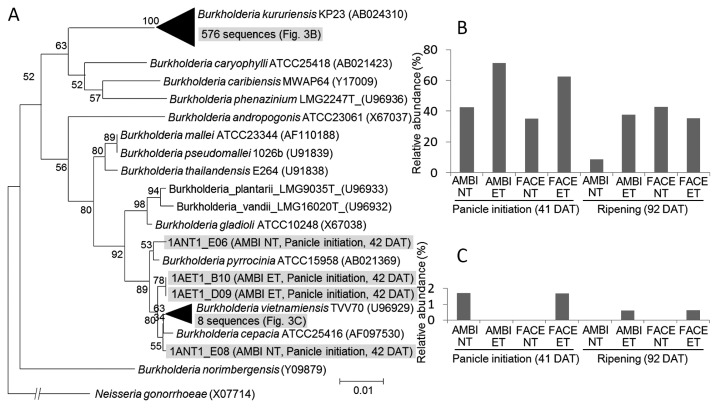
Phylogenetic position and distribution of rice root–associated *Burkholderia*. (A) Phylogenetic tree of rice root–associated *Burkholderia* extracted from the clone libraries. (B, C) Distributions of reads belonging to the two large groups in panel (A). Accession numbers are shown following the strain names.

*Burkholderia* was the most dominant genus in all samples, except AMBI-NT at 92 DAT, (Table 3). Members of this genus differ in terms of their effects on rice plants by exhibiting pathogenic (*e.g.*, *Burkholderia glumae*) (11) or symbiotic (*e.g.*, *Burkholderia kururiensis*) interactions (21). A phylogenetic tree was constructed using 16S rRNA reads assigned to rice root–associated *Burkholderia* in the clone libraries (Fig. 3A). Most of the sequences clustered into one group that was phylogenetically close to *B. kururiensis (*Figs. 3A, B). This species was reported to colonize rice roots and significantly enhance rice growth by fixing nitrogen and producing the phytohormone auxin (21). Previous rice FACE experiments showed that the nitrogen concentration of rice was decreased by elevations in [CO_2_] (29), which suggested that nitrogen availability is a limiting factor in an elevated [CO_2_] environment. In the present study, the relative abundance of *Burkholderia* was still high at 92 DAT in under the elevated [CO_2_] condition (Table 3). These results suggest that when a nitrogen deficiency occurs in rice, the high relative abundance of *Burkholderia* is maintained for a longer period in order to support growth. The high relative abundance of *Burkholderia* was also maintained at 92 DAT in the ET condition (Table 3). Root–associated *Burkholderia* may also play roles to support the growth of rice stimulated by elevations in temperature (33).

A high relative abundance of *Clostridia* was observed in all treatments at 92 DAT (5.9–13.6%) (Table 3), suggesting the presence of an anaerobic environment in rice roots at the ripening stage. At 92 DAT, *Clostridium* cluster III was the most abundant genus in *Clostridia*, the members of which produce cellulosomes (highly active cellulolytic and xylanolytic complexes) (24). Plant biomass decomposed by *Clostridium* cluster III and other microbes may be a substrate for rice root–associated methanogenic *archaea* (15, 18).

One large OTU (OTU164), which was an unclassified bacterium showing 89% similarity to *Nitratiruptor tergarcus*, was abundant in AMBI-NT (33.9%) and FACE-ET (18.5%) at 92 DAT (Table 3). *Nitratiruptor tergarcus* is an *Epsilonproteobacteria* and a nitrate-reducing chemolithoautotroph (22). This OTU member is one of the main reasons why AMBI-NT and FACE-ET at 92 DAT were clustered with each other in the principal coordinate analysis (Fig. 1). The relative abundance of OTU164 was markedly decreased by the elevation in temperature (1.2% vs. 33.9% in AMBI-NT) or [CO_2_] (2.5%), but was only slightly decreased by the simultaneous elevation in temperature and [CO_2_] (18.5%) (Table 3). These results suggested that the effects of temperature and [CO_2_] elevations interact with each other and changes in the community structure are compensated.

*Achromobacter*, a genus of sulfur-oxidizing bacteria in rice paddy fields (10), was only observed at 41 DAT (Table 3). Sulfur-oxidizing bacteria are able to oxidize reduced sulfur compounds (H_2_S, thiosulfate, and sulfite). Sulfate is the main sulfur compound that rice roots can take up (20). *Achromobacter* may play an important role in the growth of rice at the panicle initiation stage.

## Conclusion

[CO_2_] enrichment (FACE), elevated soil & water temperature (warming), and rice growth stages markedly affected the microbial communities of rice root-associated bacteria including *Methylosinus* sp., *Methylocystis* sp., *Burkholderia kururiensis*, *Bradyrhizobium* sp., *Clostridium* sp., and an unknown genus (OTU164) close to *Epsilonproteobacteria*. Most of these bacteria play important roles in the metabolism of C and N in the environment through, for example, nitrogen fixation and methane oxidation. The results of the present study will contribute to improving our understanding of microbe-mediated CN dynamics in paddy rice fields under a changing climate.

## Figures and Tables

**Table 1:** DDBJ accession numbers of 16S rRNA gene sequences

Sample	Panicle initiation stage (41 DAT)	Ripening stage (92 DAT)
AMBI-NT	AB836880–AB837055	AB837585–AB837749
AMBI-ET	AB837056–AB837234	AB837750–AB837919
FACE-NT	AB837235–AB837406	AB837920–AB838080
FACE-ET	AB837407–AB837584	AB838081–AB838242

DAT, days after transplanting; AMBI, ambient CO_2_; FACE, free-air CO_2_ enrichment; NT, normal soil and water temperature; ET, elevated soil and water temperature.

**Table 2:** Statistical characteristics of 16S rRNA gene clone libraries derived from rice roots

Rice growth stage	Panicle initiation stage (41 DAT)				Ripening stage (92 DAT)			
		
CO_2_	AMBI		FACE		AMBI		FACE	
				
Temperature	NT	ET	NT	ET	NT	ET	NT	ET
Statistics								
No. of sequences	176	179	172	178	165	170	161	162
No. of OTUs (≥97% identity)	28	22	27	28	52	45	48	32
No. of singletons	20	15	19	20	35	28	30	17
Library coverage (%)^a^	88.6	91.6	89.0	88.8	78.8	83.5	81.4	89.5
Diversity indexes								
Chao1	91.3	57.0	69.8	75.5	118.1	82.8	96.3	66.0
Shannon	2.2	1.5	2.1	1.9	3.3	3.0	3.1	2.7

DAT, days after transplanting; AMBI, ambient CO_2_; FACE, free-air CO_2_ enrichment; NT, normal soil and water temperature; ET, elevated soil and water temperature; OTU, operational taxonomic unit.

a Coverage calculated as *C_x_* = 1 – (*n_x_*/*N*), where *n_x_* is the number of singletons that are encountered only once in a library and *N* is the total number of clones.

**Table 3:** Phylogenetic compositions of 16S rRNA gene clone libraries derived from rice roots (values are relative abundances as a percentage of total bacteria)

Rice growth stage	Panicle initiation stage (41 DAT)				Ripening stage (92 DAT)			
		
CO_2_	AMBI		FACE		AMBI		FACE	
				
Temperature	NT	ET	NT	ET	NT	ET	NT	ET
*Proteobacteria*								
*Alphaproteobacteria*	10.8	3.4	5.2	5.6	17.6	26.5	15.5	12.3
*Methylosinus* (A)	0.0	0.0	0.0	0.0	6.1	11.2	2.5	4.3
*Methylocystis* (B)	0.0	0.6	0.0	1.1	3.0	4.1	2.5	2.5
(A)+(B)	0.0	0.6	0.0	1.1	9.1	15.3	5.0	6.8
*Bradyrhizobium*	0.0	0.6	1.2	1.1	5.5	4.1	5.6	4.9
*Rhizobium*	10.2	2.2	4.1	1.7	1.8	4.7	1.9	0.0
*Betaproteobacteria*	77.8	87.2	77.9	84.3	10.9	42.4	47.8	35.8
*Burkholderia*	44.9	72.1	34.9	64.0	8.5	37.6	42.9	35.2
*Ralstonia*	27.8	13.4	39.5	15.7	2.4	3.5	4.3	0.6
*Achromobacter*	4.0	1.7	2.3	3.9	0.0	0.0	0.0	0.0
*Gammaproteobacteria*	2.3	1.1	3.5	2.8	5.5	2.4	2.5	6.2
*Aquicella*	0.6	0.6	1.2	1.7	5.5	1.8	2.5	5.6
*Deltaproteobacteria*	0.0	0.0	0.0	0.0	1.2	0.6	0.0	0.0
*Firmicutes*								
*Bacilli*	1.1	0.0	0.6	0.6	0.0	0.0	0.0	0.0
*Clostridia*	1.7	0.0	1.2	1.1	9.7	5.9	11.2	13.6
*Clostridium III*	0.0	0.0	0.0	0.0	7.3	3.5	3.1	9.9
*Clostridium sensu stricto*	0.0	0.0	0.6	0.6	0.0	0.0	3.1	1.2
Others	1.1	2.2	0.6	0.6	1.8	1.8	2.5	0.6
Unclassified	5.1	6.1	11.0	5.1	53.3	20.6	20.5	31.5
Unclassified OTU164	0.0	0.0	0.0	0.0	33.9	1.2	2.5	18.5

DAT, days after transplanting; AMBI, ambient CO_2_; FACE, free-air CO_2_ enrichment; NT, normal soil and water temperature; ET, elevated soil and water temperature.

**Table 4:** Seasonal and total CH_4_ emission from each rice paddy treatment

	AMBI-NT	FACE-NT	AMBI-ET	FACE-ET
13 DAT (7 June)	0.64 ± 0.16	0.81 ± 0.16	0.64 ± 0.19	0.51 ± 0.42
27 DAT (21 June)	1.88 ± 0.81	2.68 ± 0.69	2.85 ± 1.68	3.47 ± 1.87
41 DAT (5 July)	7.85 ± 1.62	10.87 ± 2.98	9.96 ± 2.82	11.98 ± 2.67
48 DAT (12 July)	14.00 ± 2.23	15.89 ± 3.38	17.26 ± 2.78	18.35 ± 3.14
62 DAT (26 July)	14.71 ± 3.97	14.39 ± 3.61	18.06 ± 4.08	18.69 ± 2.40
69 DAT (2 August)	15.01 ± 7.80	16.25 ± 4.15	15.42 ± 3.77	18.37 ± 5.09
76 DAT (9 August)	30.36 ± 8.50	22.36 ± 6.44	27.50 ± 7.63	27.16 ± 6.63
83 DAT (16 August)	22.98 ± 4.55	19.96 ± 5.44	26.72 ± 9.12	31.83 ± 8.68
90 DAT (23 August)	11.71 ± 5.70	14.96 ± 10.29	13.30 ± 4.62	14.33 ± 9.67
Total emission (41–90 DAT)	20.36 ± 5.04	19.64 ± 4.69	22.22 ± 2.99	24.13 ± 3.33

ANOVA results				
[CO_2_]	not significant (*P*=0.67)			
Temperature	*P*=0.05			
[CO_2_]×Temperature	not significant (*P*=0.34)			

Data are the mean ± SD of four replicated plots (except *n* = 3 for AMBI-ET at 62 DAT and FACE-ET at 83 DAT).

Seasonal CH_4_ emissions are shown in mg C-CH_4_ m^−2^ h^−1^ and total CH_4_ emission in g C-CH_4_ m^−2^.

Total emission shows the cumulative emission of CH_4_ during 41–90 DAT, which almost corresponded to the two microbial sampling dates.

DAT, days after transplanting; AMBI, ambient CO_2_; FACE, free-air CO_2_ enrichment; NT, normal soil and water temperature; ET, elevated soil and water temperature.
